# Assessment of Anti-Hypertensive Drug Adherence by Serial Aldosterone-To-Renin Ratio Measurement

**DOI:** 10.3389/fphar.2021.668843

**Published:** 2021-05-10

**Authors:** Fabrizio Buffolo, Elisa Sconfienza, Jacopo Burrello, Isabel Losano, Giulio Mengozzi, Gabriella Priolo, Valeria Avataneo, Antonio D’Avolio, Franco Veglio, Franco Rabbia, Paolo Mulatero, Silvia Monticone

**Affiliations:** ^1^ Division of Internal Medicine and Hypertension Unit, Department of Medical Sciences, University of Torino, Torino, Italy; ^2^ Department of Laboratory Medicine, AOU Città della Salute e della Scienza, Turin, Italy; ^3^ Laboratory of Clinical Pharmacology and Pharmacogenetics, Department of Medical Sciences, University of Turin, Amedeo di Savoia Hospital, Turin, Italy

**Keywords:** drug adherence, angiotensin receptor blocker, angiotensin-converting enzyme inhibitor, anti-hypertensive treatment, aldosterone, renin, therapeutic drug monitoring

## Abstract

Reduced or absent compliance to anti-hypertensive treatment is a major obstacle to the achievement of blood pressure target in patients with arterial hypertension. Current available methods for therapeutic adherence assessment display low accuracy, limited applicability in clinical practice and/or high costs. We designed a prospective study to evaluate the accuracy of serial measurement of ARR to assess the therapeutic compliance to RAAS inhibitors. We prospectively enrolled 80 subjects: 40 patients with arterial hypertension and 40 normotensive controls. The ARR was evaluated at baseline and 2 and 8 week after initiation of a RAAS inhibitor in patients with hypertension, and at baseline and 2 weeks for the control group. Adherence to the prescribed therapy was confirmed by therapeutic drug monitoring. We observed a significant increase of renin levels and reduction of aldosterone levels after RAAS inhibitors initiation, with consequent reduction of ARR. Delta ARR (ΔARR), defined as relative change in ARR before and after treatment initiation, provided high accuracy for determination of therapeutic compliance, with an AUC of 0.900 at 2 weeks and 0.886 at 8 weeks. A cut-off of −48% of ΔARR provided 90% sensitivity and 75% specificity, at 2 and 8 weeks. In conclusion, the measurement of ΔARR is a powerful test, cheap and widely available to accurately identify the non-adherence to RAAS inhibitors treatment. Herein we propose the implementation of ΔARR in clinical practice through a multi-step flow-chart for the management of patients with uncontrolled blood pressure, with identification of those suspected of non-adherence, reserving therapeutic drug monitoring for non-adherence confirmation.

## Introduction

Partial or absent adherence to medical treatment hinders the achievement of therapeutic goals in chronic diseases treatment and control of cardiovascular risk factors, including arterial hypertension, dyslipidemia and diabetes (Fischer et al., 2010). In the last three decades, the awareness of arterial hypertension increased in high-income countries, together with increasing rate of prescription of anti-hypertensive treatment (NCD Risk Factor Collaboration (NCD-RisC), 2019). However, achievement of blood pressure control is still scant, ranging from 29 to 58% (NCD Risk Factor Collaboration (NCD-RisC), 2019). Uncontrolled blood pressure is the consequence of several factors: inadequate anti-hypertensive treatment, medical inertia, poor-adherence to dietary recommendations and medical prescriptions (Williams et al., 2018). About 40% of patients with hypertension are non-adherent or partially adherent to prescribed medications, and the prevalence raises to ∼80% among patients with uncontrolled blood pressure (Abegaz et al., 2017).

Poor compliance to anti-hypertensive prescriptions has been associated with increased risk of cardiovascular events (Corrao et al., 2011), heart failure (Perreault et al., 2009), hemorrhagic and ischemic stroke (Kettani et al., 2009; Xu et al., 2017). In this context, ESC/ESH guideline of 2018, reinforced recommendations for screening patients adherence, especially when blood pressure control is not achieved (Williams et al., 2018). Early recognition of reduced compliance can avoid novel inappropriate prescriptions and mitigate the cost of unnecessary investigations (Williams et al., 2018).

Several methods have been proposed for assessment of therapeutic adherence (Burnier and Egan, 2019). However, in everyday practice detection of non-adherent patients is still a challenge. Clinical interview, although considered useful by many clinicians, is highly unreliable (Meddings et al., 2012). Questionnaires tend to overestimate patients adherence (Prado et al., 2007; Pandey et al., 2015) and are preferentially used in clinical studies (Burnier and Egan, 2019). Pill count and prescription refill data are helpful for epidemiological reasons, but less useful in clinical practice. Electronic monitoring systems are packages of drugs equipped with microcircuit able to detect and record removal of the pills. The system guarantees 97% accuracy, but its application in clinical routine is unrealistic (Christensen et al., 2009). Nowadays, TDM by liquid-chromatography tandem mass spectrometry from urine or blood specimens is considered the gold standard by ESC/ESH guideline (Williams et al., 2018). Major limitations of TDM are the cost, the limited distribution of adequately equipped laboratories and white coat adherence (Burnier and Egan, 2019).

ARB and ACEi are recommended as first line therapy for uncomplicated arterial hypertension by ESC/ESH guideline (Williams et al., 2018). As result of the inhibition of RAAS, ARB, and ACEi reduce serum aldosterone and increase renin, with consequent reduction of ARR (Mulatero et al., 2002; Viola et al., 2015). In this context, we designed a prospective study to evaluate the use ARR changes, before and after RAAS inhibitors initiation, as means to assess adherence to ARB or ACEi prescription.

## Materials and Methods

### Study Design

The study was conducted in accordance with Declaration of Helsinki and the protocol of the study was approved by our local ethic committee. All recruited patients agreed to participate to the study by written informed consent.

In the Evaluation of TReatment adherence Using Renin and aldosterone ImmunoAssay (ETRURIA) study, we prospectively enrolled 80 patients (40 patients with arterial hypertension and 40 healthy controls) referred to the Hypertension Unit of the University of Torino, Italy.

Inclusion criteria for patients with hypertension were 1) diagnosis of arterial hypertension according with ESC/ESH guideline (Williams et al., 2018), 2) age between 18 and 80 year-old. Exclusion criteria were 1) patients treated with beta-blockers, clonidine, diuretics or RAAS inhibitors and 2) a secondary cause of arterial hypertension (including primary aldosteronism (PA), pheochromocytoma, hypercortisolism, hyperthyroidism, and renovascular hypertension). Exclusion criteria for controls were 1) antihypertensive treatment both at recruitment time and/or during follow-up and 2) chronic treatment with non-steroidal anti-inflammatory drugs and contraceptive pill.

At recruitment, patients underwent complete visit, BP assessment through three consecutive BP measurements in the sitting position, according to the 2018 ESC/ESH guideline (Williams et al., 2018), collection of blood and urine samples for renin and aldosterone assays. After recruitment visit, one of three different RAAS inhibitors, telmisartan, olmesartan or ramipril was prescribed. The choice of specific drug and dosage was left to treating physician, according to respective preferences and individual clinical aspects.

**FIGURE 1:**
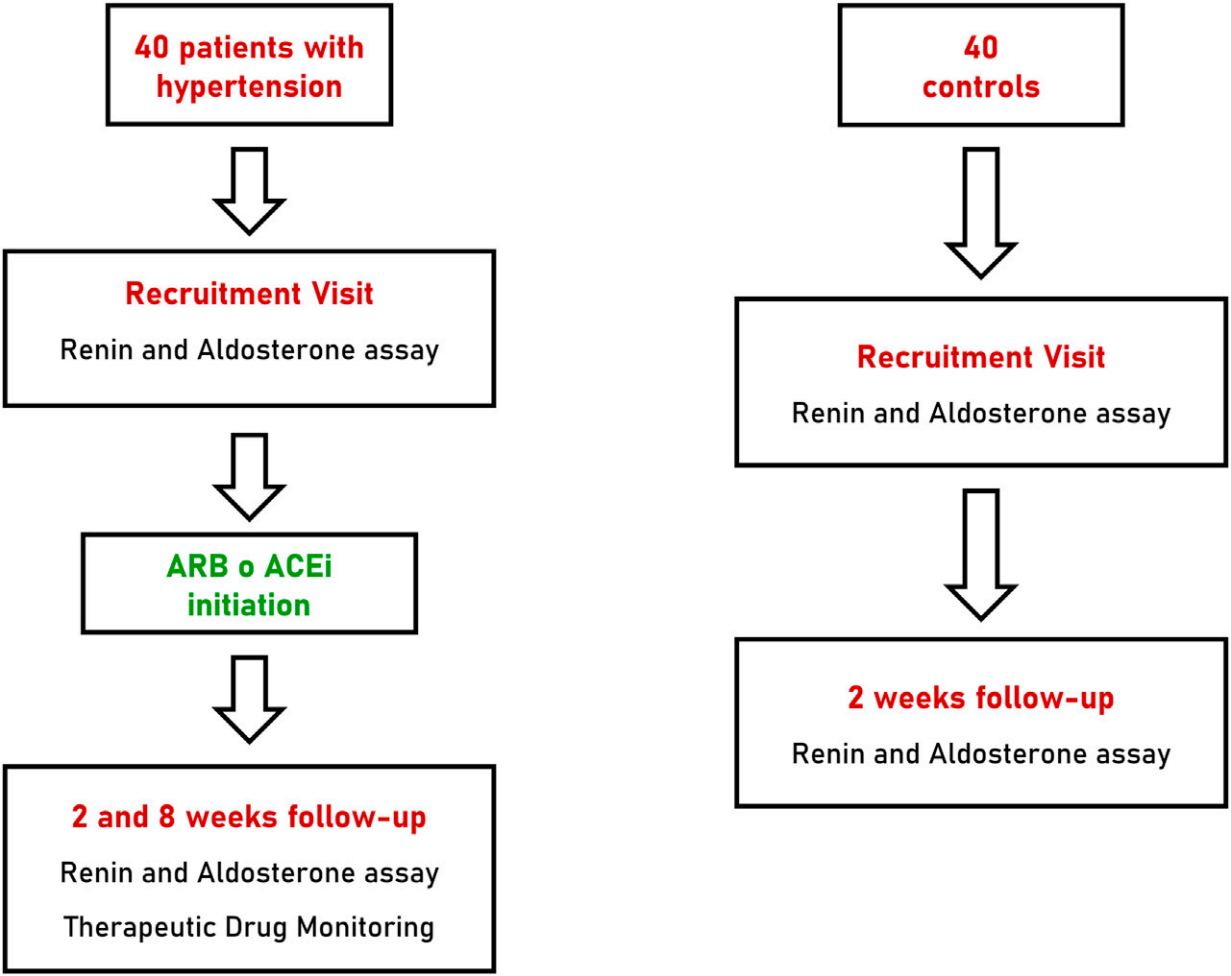
Flow-chart of recruitment, treatment and follow-up of patients with hypertension and controls. ARB, angiotensin receptor blocker; ACEi, angiotensin converting enzyme inhibitor.

Patients with arterial hypertension underwent two follow-up visits at 2 and 8 weeks after initiation of anti-hypertensive treatment with a RAAS inhibitor. At each follow-up visit all patients underwent blood pressure measurements as reported above and collection of blood and urine specimens for biochemical assays and TDM. Patients of the control group underwent a single follow-up visit, 2 weeks after recruitment, with collection of blood samples for renin and aldosterone measurements (Figure 1).

### Biochemical Measurements and Therapeutic Drug Monitoring

Blood samples for aldosterone concentration were collected into room temperature serum tubes and for direct renin into room temperature plasma EDTA tubes. Tubes were then centrifuged (3000 rpm, 15 min, 27–28°C) and frozen at −20°C. Aldosterone and direct renin were measured with a fully automated chemiluminescent immunometric method (LIAISON®; DiaSorin, Saluggia, Italy); ARR was calculated at recruitment, and after 2 and 8 weeks of treatment. Blood samples were collected after 15 min of sitting position, as recommended by ESH consensus (Mulatero et al., 2020) and Endocrine Society guideline (Funder et al., 2016) for primary aldosteronism.

Dosage of therapeutic drugs in plasma and urine specimens was performed to assess compliance to prescribed anti-hypertensive treatment at 2 and 8 weeks after initiation of ARB or ACEi. Plasma for drug monitoring was collected in lithium heparin tubes. Measurements of therapeutic drugs in urine and plasma was performed by Ultra High-Performance Liquid Chromatography Mass Spectrometry as previously detailed (De Nicolò et al., 2016; De Nicolò et al., 2017). Olmesartan and ramipril presence were assessed in both urine and plasma, telmisartan was assessed exclusively in plasma specimens.

### Statistical Analysis

IBM SPSS Statistics version 26.0 (IBM Corp., Armonk, NY, United States) and PRISM software (GraphPad, San Diego, CA, United States) were used for statistical analysis and graph preparation.

Variables were considered parametric or non-parametric according to their distribution. Continuous variables with normal distribution are expressed as mean ± standard deviation. Variables with non-normal distribution are expressed as median (interquartile range). Mann Whitney U test was applied for non-normally distributed data for independent samples and Wilcoxon signed-rank test for paired samples. ΔARR and delta-SBP (ΔSBP) were defined as percentage variation of ARR and SBP before and after initiation of ARB or ACEi treatment and were calculate both at 2 and 8 weeks. To assess the diagnostic accuracy of ΔARR to predict patient adherence, we used ROC curves. ROC curves are the graphical representation of the combination of true positive results (sensitivity) and false positive results (1- specificity) for a discrete number of different thresholds. The area between the ROC curve and the diagonal is the AUC. The AUC is thus a metric for the ability of a test, score or model to discriminate between individuals with and without a disease, a characteristic or, in this case, between patients assuming or not RAAS inhibitors. The greatest is the ROC curves, the highest is the discriminatory capacity of a test (Janssens and Martens, 2020). Optimized sensitivity was defined by the maximum sensitivity with specificity non inferior to 75%. Intraindividual coefficients of variation was calculated as the standard deviation divided by the mean value.

A surrogate combined index of ΔARR and ΔSBP was created by linear weighted combination of ΔARR and ΔSBP. ROC curves were then used to assess the accuracy of ΔSBP alone and in combination with ΔARR.

## Results

### Cohort Descriptive Statistics

A total of 80 subjects were prospectively recruited for the ETRURIA Study in Torino: 40 patients with arterial hypertension and 40 controls (Table 1). Of the 40 patients with arterial hypertension, 24 (60.0%) started a new anti-hypertensive treatment with telmisartan, 12 (30.0%) with olmesartan and 4 (10.0%) with ramipril. Baseline values of renin (12.5 [5.9–26.8] *vs.* 12.0 [6.3–15.0] µU/ml, *p* = 0.102), aldosterone (11.5 [8.7–16.0] *vs.* 12.8 [9.1–18.3] ng/dl, *p* = 0.292) and ARR (0.79 [0.53–1.95] *vs.* 1.14 [0.72–2.60] ng/dl/µU/ml, *p* = 0.100) were similar in the cohort treated with RAAS inhibitors and control group, respectively.

**TABLE 1:** Descriptive statistics.

	Treatment with RAAS inhibitor (*n* = 40)	Control Group (*n* = 40)
Age	50 ± 11	37 ± 15
Sex		
Male, *n* (%)	30 (75.0)	21 (52.5)
Female, *n* (%)	10 (25.0)	19 (47.5)
SBP (mmHg)	144 ± 13	124 ± 21
DBP (mmHg)	88 ± 10	76 ± 14
Creatinine (mg/dl)	0.89 ± 0.14	0.79 ± 0.10
Sodium (mmol/L)	140.4 ± 1.7	139.6 ± 2.4
Potassium (mmol/L)	4.1 ± 0.4	4.0 ± 0.3
Renin (µU/ml)	12.5 (5.9–26.8)	12.0 (6.3–15.0)
Aldosterone (ng/dl)	11.5 (8.7–16.0)	12.8 (9.1–18.3)
ARR (ng/dl/µU/ml)	0.79 (0.53–1.95)	1.14 (0.72–2.60)
RAAS inhibitor		–
Telmisartan	24 (60)	
Olmesartan	12 (30)	
Ramipril	4 (10)	

Values are mean ± SD, median (IQR), or absolute number (%). Comparisons were performed by Student *t* test for normally distributed continuous variables, Mann Whitney U test for non-normally distributed continuous variables and χ2 for categorical variables. RAAS, renin-angiotensin-aldosterone system; SBP, systolic blood pressure; DBP, diastolic blood pressure; ARR, aldosterone-to-renin ration.

### Hormonal Changes at Follow up

**FIGURE 2:**
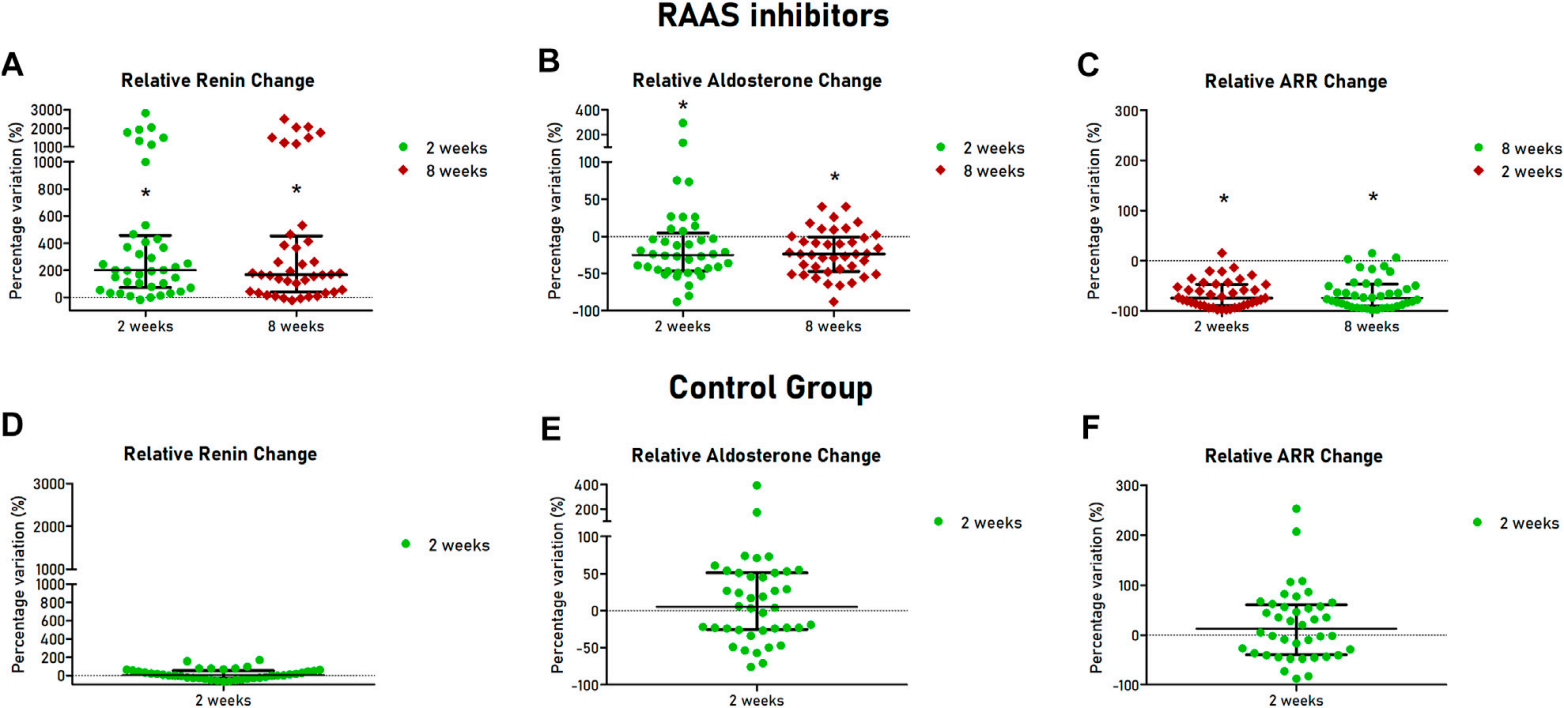
Hormonal changes at follow-up visits of patients with hypertension and controls. Scatter dot plots are showed with median (central line) and 25th–75th interquartile range (extremities). For patients treated with RAAS inhibitors relative changes of renin **(A)**, aldosterone **(B)** and ARR **(C)** are showed in the upper part of the Figure. For healthy controls, relative changes of renin **(C)**, aldosterone **(D)** and ARR **(E)** are showed in the lower part of the Figure. RAAS, renin-angiotensin-aldosterone system; ARR, aldosterone-to-renin ratio.

All patients completed the 8 weeks follow up. After 2 and 8 weeks, assumption of ARBs or ACEi was confirmed by TDM in all patients of the cohort treated with RAAS inhibitors. Renin increased after 2 and 8 weeks after initiation of ACEi or ARBs (12.5 [5.9–26.8] *vs.* 49.1 [14.6–118.0] µU/ml at 2 weeks, *p* < 0.001 and 36.5 [15.1–104.9] µU/ml at 8 weeks, *p* < 0.001), with median relative change of +200% and + 167% at 2 and 8 weeks respectively (Figure 2A). Aldosterone was reduced at 2 and 8 weeks in the treated cohort (11.5 [8.7–16.0] *vs.* 8.9 [7.0–11.2] ng/dl at 2 weeks, *p* = 0.002 and 8.8 [6.4–11.7] ng/dl at 8 weeks, *p* < 0.001), with median relative change of −25 and −24% at 2 and 8 weeks, respectively, (Figure 2B). Consequently, ARR decreased after 2 and 8 weeks of treatment (0.79 [0.53–1.95] *vs.* 0.17 [0.07–0.58] ng/dl/µU/ml at 2 weeks, *p* < 0.001 and 0.16 [0.10–0.64] ng/dl/µU/ml at 8 weeks, *p* < 0.001), with median relative change of −75 and −74% at 2 and 8 weeks, respectively (Figure 2C).

As expected, in the control group, after 2 weeks, renin (12.0 [6.3–15.0] *vs.* 13.6 [5.9–17.5] µU/ml, *p* = 0.301) and aldosterone (12.8 [9.1–18.4] *vs.* 12.5 [8.8–19.8] ng/dl, *p* = 0.752) were not significantly different from baseline values (Figures 2D,E). Similarly, no significant changes of ARR were observed (1.14 [0.72–2.60] *vs.* 1.11 [0.56–3.12] ng/dl/µU/mi, *p* = 0.237) (Figure 2F). Intraindividual mean variability of aldosterone levels, defined as coefficient of variation, was 30%, similar to figures reported in a recent study (Yozamp et al., 2021).

### Diagnostic Performances of ΔARR

**FIGURE 3:**
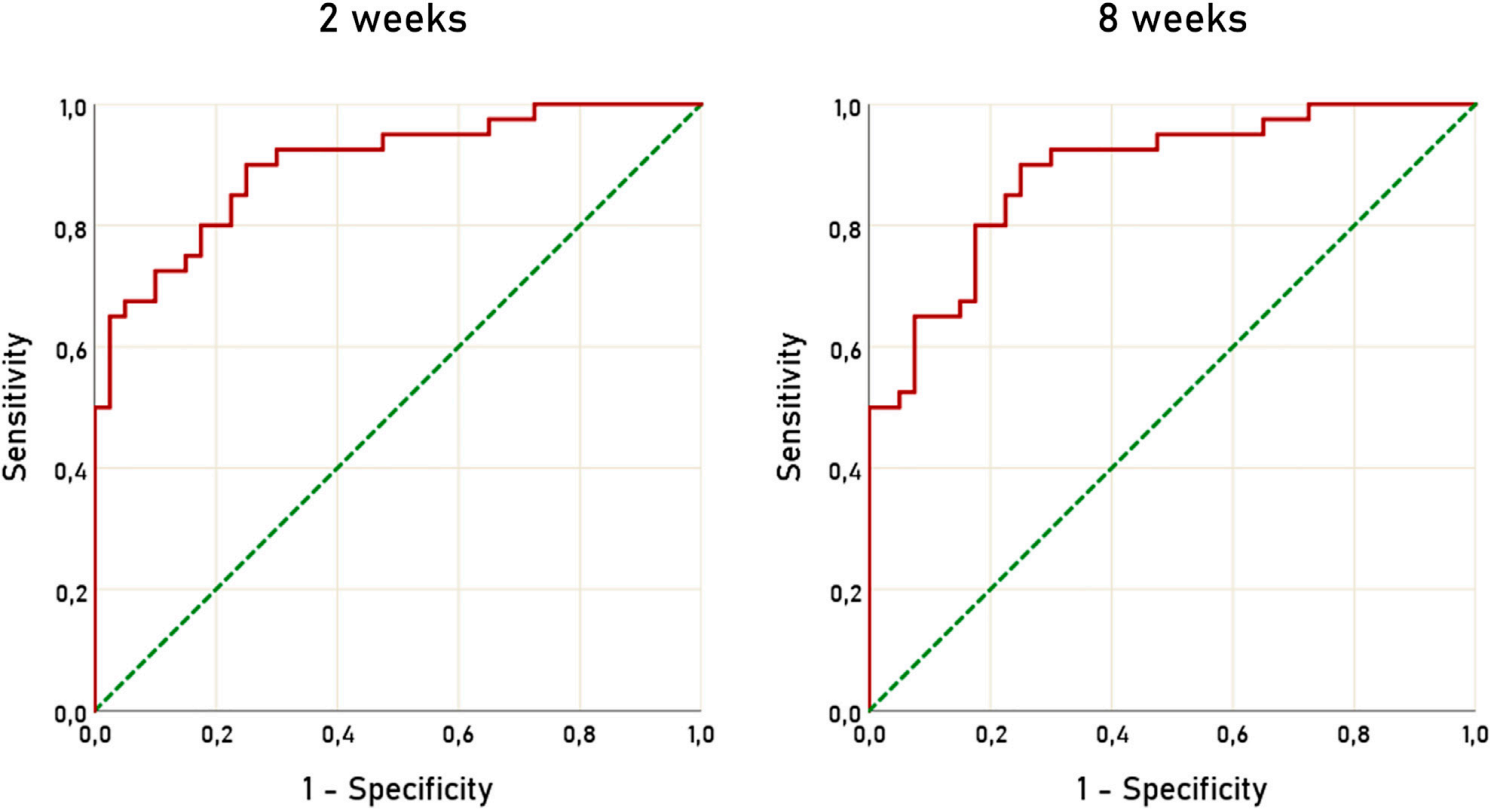
ROC curves of ΔARR for prediction of non-adherence to treatment with RAAS inhibitors. ROC, receiver operating characteristic; ΔARR, delta aldosterone-to-renin ratio; RAAS, renin-angiotensin-aldosterone system.

To assess the diagnostic accuracy of ΔARR to detect patients not compliant to ARB or ACEi we used ROC curves, at 2 and 8 weeks after RAAS inhibitors initiation. The AUC for ΔARR at 2 weeks was 0.900 (95% C.I. 0.834–0.966) and 0.886 (95% C.I. 0.815–0.957) (Figure 3). Considering ΔARR as screening test for non-adherence detection, we selected the cut-off of −48% to optimize ΔARR sensitivity. With the threshold of −48%, ΔARR displayed a sensitivity of 90% and specificity of 75%, both at 2 and 8 weeks. The correspondent accuracy was 82.5%, both at 2 and 8 weeks. On the opposite, a threshold of ΔARR + 15% provided 100% specificity and 50% sensitivity and 75% accuracy.

In order to assess whether the blood pressure response to RAAS inhibitors could improve the accuracy of ΔARR alone, we analyzed the diagnostic performance of ΔSBP alone and in combination with ΔARR, by means of a surrogate index created with linear weighted combination of ΔARR and ΔSBP (Supplementary Figure S1). We observed a moderate predictive performance of ΔSBP, with AUC of 0.791 and 0.795 at 2 and 8 weeks, respectively. However, the combination of ΔARR and ΔSBP did not significantly increase the diagnostic performance, compared to ΔARR alone (AUC 0.900 vs. 0.891, *p* = 0.863 at 2 weeks; AUC 0.886 vs. 0.888, *p* = 0.970).

**FIGURE 4:**
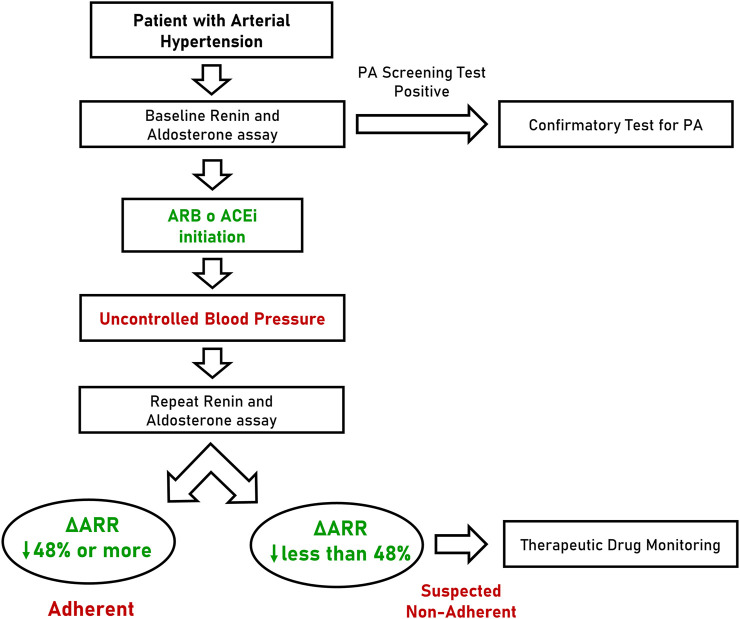
Flow-chart proposal for the management of patients with arterial hypertension. PA, primary aldosteronism; ARB, angiotensin receptor blocker; ACEi, angiotensin converting enzyme inhibitor; ΔARR, delta aldosterone-to-renin ratio.

Considering the performance of ΔARR, we hypothesized a flow-chart for the management of patients with arterial hypertension (Figure 4). In this scenario, the ARR is determined for the first time at diagnosis or during screening test for PA (when interfering drugs are usually withdrawn) and followed by the prescription of anti-hypertensive therapy that, according to ESC/ESH guideline (Williams et al., 2018) will include an inhibitor of RAAS for most patients. In case of uncontrolled blood pressure, during follow-up visits or whenever non-adherence is suspected, clinicians may prescribe the repetition of renin and aldosterone assay. In case of reduction of ARR greater than 48%, the patients can be considered, with high confidence, adherent to prescribed therapy. In contrast, when ΔARR is reduced less than 48% or increased, the patient should be suspected to be non-adherent and TDM can be proposed. Finally, when ΔARR is increased more than 15%, the chance that the patient is compliant to anti-hypertensive treatment with RAAS inhibitors is substantially null.

## Discussion

Suboptimal adherence to antihypertensive treatment is a key contributor to uncontrolled blood pressure and is associated with unfavorable outcomes (Burnier and Egan, 2019). As of today, several methods have been developed to address therapeutic adherence, but they are costly, time consuming and better suited in the context of a trial rather than the everyday clinical practice. Some authors proposed the use of renin/plasma renin activity ratio to determine adherence to aliskiren, an oral renin inhibitor, but its application was never confirmed by definitive studies (Gosse et al., 2011). However, no study previously investigated the use of ARR for determination of therapeutic compliance. In the ETRURIA study we proposed for the first time the serial ARR determination, with ΔARR measurement, as an innovative, cheap and powerful method to predict adherence in patients taking ARBs or ACEis. The application of the ΔARR to the clinical practice could be of value to select patients who are particularly suitable candidates for TDM, because of a high probability of being non adherent.

Adherence can be categorized in three major components: initiation, implementation and discontinuation (Vrijens et al., 2012). *Initiation* has been defined as the time from prescription to the assumption of the first dose. *Implementation* is the extent of time in which the prescribed dose corresponds to the dose actually taken by the patient. *Discontinuation* is the interruption of the therapy intake and *persistence* is the extent of time between *initiation* and *discontinuation*. Non-initiation is the first obstacle for adherence to anti-hypertensive treatment, involving up to 26% of patients (Fischer et al., 2011). Discontinuation rate progressively raises during follow-up, from 33% after 6 months, up to 50% at 5 years (Corrao et al., 2008). Use of ΔARR allows detection of both non-initiation and discontinuation. In fact, the changes of ARR are evident 2 weeks after treatment initiation, with similar performances at 8 weeks. Therefore, ΔARR can be applied shortly after ARBs and ACEis prescription to assess initiation of anti-hypertensive therapy. Later, ΔARR can be repeated whenever lack of persistence is suspected (e.g., in case of uncontrolled blood pressure).

Over the last years, the measurement of drug levels in blood to assess adherence has gained increasing popularity. A strategy of repeated control of anti-hypertensive adherence by TDM has showed to improve blood pressure control, by reduction of ∼19 mmHg of SBP and ∼7 mmHg of DBP (Gupta et al., 2017). However, the use of TDM by liquid-chromatography tandem-mass spectrometry as part of routine clinical care is hampered by costs, the paucity of dedicated laboratories and qualified personnel and it is therefore unfeasible for a large-scale strategy. On the contrary, ΔARR can be easily assessed by automated chemiluminescent immunometric methods, reducing the costs and amplifying the number of adequately equipped laboratories.

Use ARBs or ACEis is recommended as first line therapy for uncomplicated hypertension by ESC/ESH guideline of 2018 (Williams et al., 2018). Moreover, RAAS inhibitors are recommended in patients with diabetes and/or microalbuminuria, for long-term renal damage protection (Williams et al., 2018). Thus, the spectrum of patients that can benefit of RAAS inhibitors monitoring is wide and includes the large majority of patients with hypertension.

As recommended by ESC/ESH guideline of 2018(Williams et al., 2018), single-pill combination therapy is one of the key strategies to increase therapeutic compliance of patients with arterial hypertension. Patients treated with single-pill combination show greater persistence than patients treated with free equivalent combination, with consequent improvement of blood pressure control (Parati et al., 2021). Following ESC/ESH recommendations, the future number of patients treated with single-pill combination will likely increase. ΔARR is technically limited to the assessment of adherence to RAAS inhibitors. However, when ARB and ACEi are prescribed in a single-pill combination therapy with a calcium channel inhibitor (that have relatively small effect on RAAS (Mulatero et al., 2002), ΔARR would allow monitoring of the whole antihypertensive therapy.

Following the results of the seminal studies of Laragh and colleagues in 70s, RAAS profiling has been considered pivotal for the classification of arterial hypertension and for anti-hypertensive treatment guidance (Brunner et al., 1972; Laragh et al., 1972). Moreover, ARR is the most reliable and widely accepted test for screening of PA. ESH consensus (Mulatero et al., 2020) and Endocrine Society guideline (Funder et al., 2016) recommend to screen for PA ∼50–60% of patients with arterial hypertension, including all patients with blood pressure ≥160/100 mmHg or ≥150/100 mmHg respectively. Recent studies progressively expanded the spectrum of PA (Brown et al., 2017; Brown et al., 2020), leading some experts to suggest screening for PA to all patients with hypertension (Vaidya and Carey, 2020). In this scenario, screening for PA should be performed in the large majority of patients with arterial hypertension, by assessment of ARR. This approach, will easily fit in the flow-chart that we propose for the management and monitoring of patient with hypertension (Figure 4). An ideal patient with newly-diagnosed arterial hypertension can undergo ARR evaluation before anti-hypertensives initiation or with non-interfering drugs (e.g., calcium-channel blockers or alpha-blockers). If PA is excluded, the results of ARR can be considered the first ARR assessment useful for ΔARR determination during clinical follow-up, when ARB or ACEi is initiated.

Limitations of the ETRURIA study are the relatively small sample size, the lack of age and sex matching, the validation of ΔARR only in patients without other drugs interfering with RAAS and the absent evaluation of ΔARR with other anti-hypertensive drugs (beyond RAAS inhibitors) alone or in combination. Additionally, we did not assess the short-term intraindividual variation of aldosterone measurement, the potassium changes at follow up and we cannot establish whether the performance of ΔARR remains similar after 8 weeks of follow up. The strengths are the prospective design of the study, the use of a selected control group to account for RAAS variation, and the double (short- and medium-term) follow-up that demonstrated similar performances, reinforcing reproducibility ΔARR accuracy.

## Conclusion

Serial assessment of renin and aldosterone by chemiluminescent immunometric assay, before and after initiation of anti-hypertensive therapy with ACEis or ARBs, is an accurate method for detection of patients non-adherent to RAAS inhibitors prescriptions. ΔARR assessment can be included in a multi-step strategy for the management of patients with arterial hypertension, that guarantees wide applicability and reduction of costs, limiting TDM to patients suspected of non-adherence after ΔARR screening.

## Data Availability

The raw data supporting the conclusion of this article will be made available by the authors, without undue reservation.

## Abbreviations

ACEi, Angiotensin-converting enzyme inhibitors; ARB, angiotensin receptor blockers; ARR, aldosterone-to-renin ration; AUC, area under the curve; DBP, diastolic blood pressure; ESC/ESH, European society of cardiology/European society of hypertension; ETRURIA, evaluation of treatment adherence using renin and aldosterone immunoassay; RAAS, renin-angiotensin-aldosterone system; ROC, receiver operating characteristics; SBP, systolic blood pressure; TDM, therapeutic drug monitoring; ΔARR, delta aldosterone-to-renin ratio; ΔSBP, delta systolic blood pressure.

## References

[B1] Abegaz T. M. Shehab A. Gebreyohannes E. A. Bhagavathula A. S. Elnour A. A. (2017). Nonadherence to Antihypertensive Drugs: A Systematic Review and Meta-Analysis. Medicine 96, e5641. 10.1097/MD.0000000000005641 28121920 PMC5287944

[B2] Brown J. M. Robinson-Cohen C. Luque-Fernandez M. A. Allison M. A. Baudrand R. Ix J. H. (2017). The Spectrum of Subclinical Primary Aldosteronism and Incident Hypertension: A Cohort Study. Ann. Intern. Med. 167, 630–641. 10.7326/M17-0882 29052707 PMC5920695

[B3] Brown J. M. Siddiqui M. Calhoun D. A. Carey R. M. Hopkins P. N. Williams G. H. (2020). The Unrecognized Prevalence of Primary Aldosteronism: A Cross-Sectional Study. Ann. Intern. Med. 173, 10–20. 10.7326/M20-0065 32449886 PMC7459427

[B4] Brunner H. R. Laragh J. H. Baer L. Newton M. A. Goodwin F. T. Krakoff L. R. (1972). Essential Hypertension: Renin and Aldosterone, Heart Attack and Stroke. N. Engl. J. Med. 286, 441–449. 10.1056/NEJM197203022860901 4257928

[B5] Burnier M. Egan B. M. (2019). Adherence in Hypertension. Circ. Res. 124, 1124–1140. 10.1161/CIRCRESAHA.118.313220 30920917

[B6] Christensen A. Osterberg L. G. Hansen E. H. (2009). Electronic Monitoring of Patient Adherence to Oral Antihypertensive Medical Treatment: a Systematic Review. J. Hypertens. 27, 1540–1551. 10.1097/HJH.0b013e32832d50ef 19474761

[B7] Corrao G. Zambon A. Parodi A. Poluzzi E. Baldi I. Merlino L. (2008). Discontinuation of and Changes in Drug Therapy for Hypertension Among Newly-Treated Patients: a Population-Based Study in Italy. J. Hypertens. 26, 819–824. 10.1097/HJH.0b013e3282f4edd7 18327094

[B8] Corrao G. Parodi A. Nicotra F. Zambon A. Merlino L. Cesana G. (2011). Better Compliance to Antihypertensive Medications Reduces Cardiovascular Risk. J. Hypertens. 29, 610–618. 10.1097/HJH.0b013e328342ca97 21157368

[B9] De Nicolò A. Avataneo V. Rabbia F. Bonifacio G. Cusato J. Tomasello C. (2016). UHPLC-MS/MS Method with Protein Precipitation Extraction for the Simultaneous Quantification of Ten Antihypertensive Drugs in Human Plasma from Resistant Hypertensive Patients. J. Pharm. Biomed. Anal. 129, 535–541. 10.1016/j.jpba.2016.07.049 27497654

[B10] De Nicolò A. Avataneo V. Rabbia F. Sciandra M. Tosello F. Cusato J. (2017). UHPLC-MS/MS Method with Sample Dilution to Test Therapeutic Adherence through Quantification of Ten Antihypertensive Drugs in Urine Samples. J. Pharm. Biomed. Anal. 142, 279–285. 10.1016/j.jpba.2017.05.018 28538203

[B11] Fischer M. A. Stedman M. R. Lii J. Vogeli C. Shrank W. H. Brookhart M. A. (2010). Primary Medication Non-adherence: Analysis of 195,930 Electronic Prescriptions. J. Gen. Intern. Med. 25, 284–290. 10.1007/s11606-010-1253-9 20131023 PMC2842539

[B12] Fischer M. A. Choudhry N. K. Brill G. Avorn J. Schneeweiss S. Hutchins D. (2011). Trouble Getting Started: Predictors of Primary Medication Nonadherence. Am. J. Med. 124, 1081.E9–1081.E22. 10.1016/j.amjmed.2011.05.028 22017787

[B13] Funder J. W. Carey R. M. Mantero F. Murad M. H. Reincke M. Shibata H. (2016). The Management of Primary Aldosteronism: Case Detection, Diagnosis, and Treatment: An Endocrine Society Clinical Practice Guideline. J. Clin. Endocrinol. Metab. 101, 1889–1916. 10.1210/jc.2015-4061 26934393

[B14] Gosse P. Coulon P. Brossaud J. Corcuff J.-B. (2011). A Simple Test to Appreciate Compliance to Aliskiren Treatment. J. Hypertens. 29, 2038. 10.1097/HJH.0b013e32834acb17 21904156

[B15] Gupta P. Patel P. Štrauch B. Lai F. Y. Akbarov A. Gulsin G. S. (2017). Biochemical Screening for Nonadherence Is Associated with Blood Pressure Reduction and Improvement in Adherence. Hypertension 70, 1042–1048. 10.1161/HYPERTENSIONAHA.117.09631 28847892 PMC5642335

[B16] Janssens A. C. J. W. Martens F. K. (2020). Reflection on Modern Methods: Revisiting the Area under the ROC Curve. Int. J. Epidemiol. 49, 1397–1403. 10.1093/ije/dyz274 31967640

[B17] Kettani F-Z. Dragomir A. Côté R. Roy L. Bérard A. Blais L. (2009). Impact of a Better Adherence to Antihypertensive Agents on Cerebrovascular Disease for Primary Prevention. Stroke 40, 213–220. 10.1161/STROKEAHA.108.522193 19038916

[B18] Laragh J. H. Sealey J. Brunner H. R. (1972). The Control of Aldosterone Secretion in Normal and Hypertensive Man: Abnormal Renin-Aldosterone Patterns in Low Renin Hypertension. Am. J. Med. 53, 649–663. 10.1016/0002-9343(72)90160-x 4342888

[B19] Meddings J. Kerr E. A. Heisler M. Hofer T. P. (2012). Physician Assessments of Medication Adherence and Decisions to Intensify Medications for Patients with Uncontrolled Blood Pressure: Still No Better Than a Coin Toss. BMC Health Serv. Res. 12, 270. 10.1186/1472-6963-12-270 22909303 PMC3570326

[B20] Mulatero P. Rabbia F. Milan A. Paglieri C. Morello F. Chiandussi L. (2002). Drug Effects on Aldosterone/plasma Renin Activity Ratio in Primary Aldosteronism. Hypertension 40, 897–902. 10.1161/01.hyp.0000038478.59760.41 12468576

[B21] Mulatero P. Monticone S. Deinum J. Amar L. Prejbisz A. Zennaro M.-C. (2020). Genetics, Prevalence, Screening and Confirmation of Primary Aldosteronism: a Position Statement and Consensus of the Working Group on Endocrine Hypertension of the European Society of Hypertension. J. Hypertens. 38, 1919–1928. 10.1097/HJH.0000000000002510 32890264

[B22] NCD Risk Factor Collaboration (NCD-RisC) (2019). Long-term and Recent Trends in Hypertension Awareness, Treatment, and Control in 12 High-Income Countries: an Analysis of 123 Nationally Representative Surveys. Lancet 394, 639–651. 10.1016/S0140-6736(19)31145-6 31327564 PMC6717084

[B23] Pandey A. Raza F. Velasco A. Brinker S. Ayers C. Das S. R. (2015). Comparison of Morisky Medication Adherence Scale with Therapeutic Drug Monitoring in Apparent Treatment-Resistant Hypertension. J. Am. Soc. Hypertens. 9, 420–426. 10.1016/j.jash.2015.04.004 26051923

[B24] Parati G. Kjeldsen S. Coca A. Cushman W. C. Wang J. (2021). Adherence to Single-Pill versus Free-Equivalent Combination Therapy in Hypertension: A Systematic Review and Meta-Analysis. Hypertension 77 (2), 692–705. 10.1161/HYPERTENSIONAHA.120.15781 33390044

[B25] Perreault S. Dragomir A. White M. Lalonde L. Blais L. Bérard A. (2009). Better Adherence to Antihypertensive Agents and Risk Reduction of Chronic Heart Failure. J. Intern. Med. 266, 207–218. 10.1111/j.1365-2796.2009.02084.x 19623691

[B26] Prado J. C. Kupek E. Mion D. (2007). Validity of Four Indirect Methods to Measure Adherence in Primary Care Hypertensives. J. Hum. Hypertens. 21, 579–584. 10.1038/sj.jhh.1002196 17443212

[B27] Vaidya A. Carey R. M. (2020). Evolution of the Primary Aldosteronism Syndrome: Updating the Approach. J. Clin. Endocrinol. Metab. 105, 3771–3783. 10.1210/clinem/dgaa606 PMC7899564 32865201

[B28] Viola A. Monticone S. Burrello J. Buffolo F. Lucchiari M. Rabbia F. (2015). Renin and Aldosterone Measurements in the Management of Arterial Hypertension. Horm. Metab. Res. 47 (6), 418–426. 10.1055/s-0035-1548868 25993253

[B29] Vrijens B. Geest S. D. Hughes D. A. Przemyslaw K. Demonceau J. Ruppar T. (2012). A New Taxonomy for Describing and Defining Adherence to Medications. Br. J. Clin. Pharmacol. 73, 691–705. 10.1111/j.1365-2125.2012.04167.x 22486599 PMC3403197

[B30] Williams B. Mancia G. Spiering W. Agabiti Rosei E. Azizi M. Burnier M. (2018). 2018 ESC/ESH Guidelines for the Management of Arterial Hypertension. Eur. Heart J. 39, 3021–3104. 10.1093/eurheartj/ehy339 30165516

[B31] Xu T. Yu X. Ou S. Liu X. Yuan J. Tan X. (2017). Adherence to Antihypertensive Medications and Stroke Risk: A Dose-Response Meta-Analysis. J. Am. Heart Assoc. 6 (7), e006371. 10.1161/JAHA.117.006371 28743788 PMC5586324

[B32] Yozamp N. Hundemer G. L. Moussa M. Underhill J. Fudim T. Sacks B. (2021). Intraindividual Variability of Aldosterone Concentrations in Primary Aldosteronism: Implications for Case Detection. Hypertension 77, 891–899. 10.1161/HYPERTENSIONAHA.120.16429 33280409 PMC7878320

