# Supplementary material for: Assessment of Anti-Hypertensive Drug Adherence by Serial Aldosterone-To-Renin Ratio Measurement
Source: Front Pharmacol. 2021 May 10;12:668843. doi: 10.3389/fphar.2021.668843 (PMC8141919; doi:10.3389/fphar.2021.668843)
Supplement: Supplementary file 1 [file Image1.pdf]

Supplementary Material

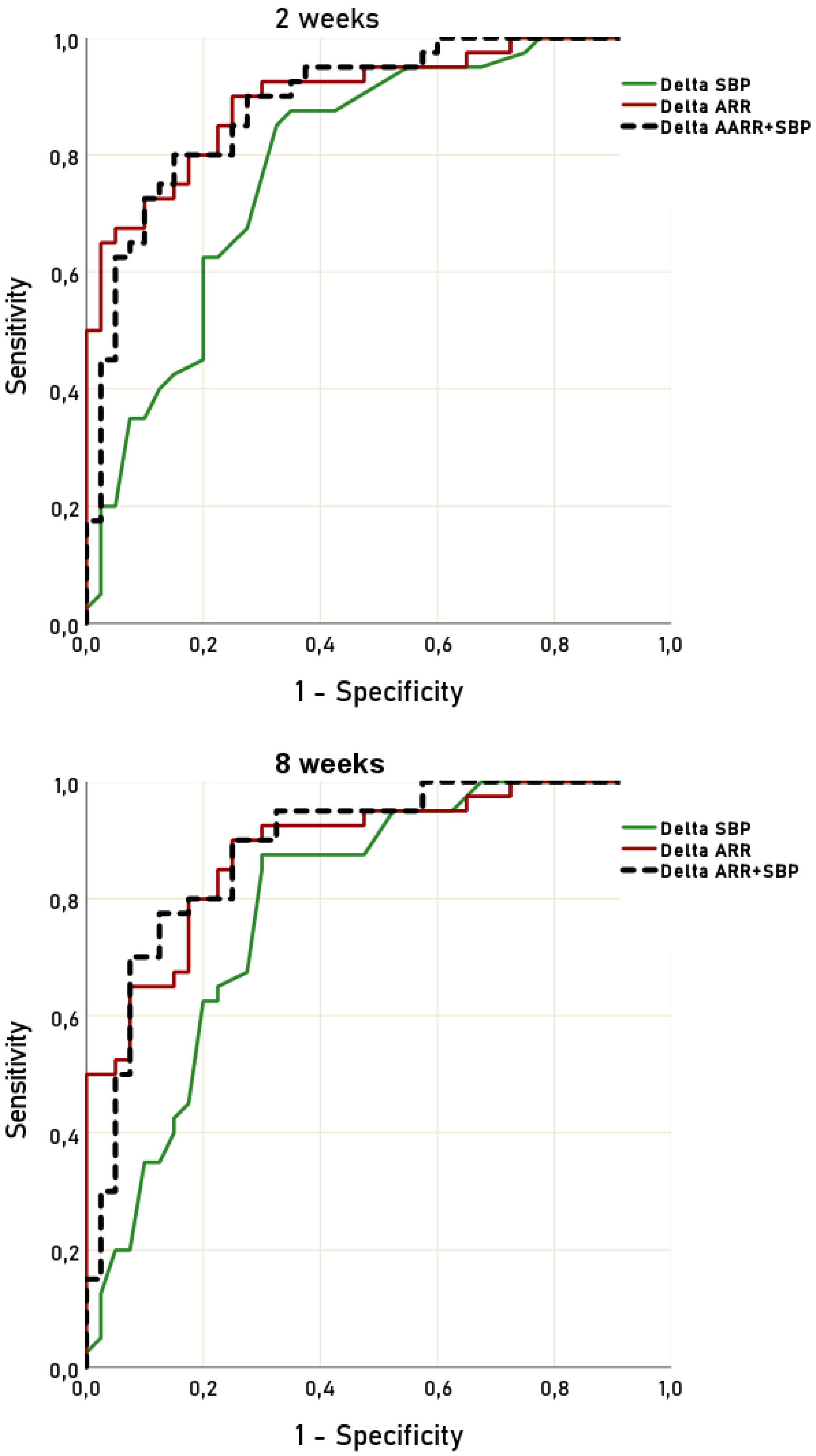

Supplementary Figure S1. ROC curves of ΔARR, ΔSBP and ΔARR+ΔSBP for prediction of non-adherence to treatment with RAAS inhibitors. ROC= receiver operating characteristic, ΔARR=delta aldosterone-to-renin ratio, SBP=systolic blood pressure, RAAS=renin-angiotensin-aldosterone system.
